# Effectiveness of Educational Videos Comparing Single Versus Multiple Topics: A Cluster Randomized Controlled Trial

**DOI:** 10.7759/cureus.64028

**Published:** 2024-07-07

**Authors:** Siti Nur Baiduri Mohd Jaini, Mohd Zulkarnain Sinor, Basaruddin Ahmad, Ruhaya Hasan, Sarliza Yasmin Sanusi

**Affiliations:** 1 Oral Health Programme, Ministry of Health Malaysia, Putrajaya, MYS; 2 Dental Public Health, School of Dental Sciences, Universiti Sains Malaysia, Kubang Kerian, MYS; 3 Nutrition, School of Dental Sciences, Universiti Sains Malaysia, Kubang Kerian, MYS; 4 Pediatric Dentistry, School of Dental Sciences, Universiti Sains Malaysia, Kubang Kerian, MYS

**Keywords:** randomized controlled trial, preschool children, oral health education, effectiveness, educational video

## Abstract

Background

Dental caries and gingivitis are preventable diseases that remain highly prevalent among children globally and, while transmissible through the transfer of oral bacteria typically from mother to child, differ from communicable diseases that are spread through direct contact, air, or vectors. Unlike communicable diseases, dental caries and gingivitis can be effectively prevented through proper oral hygiene practices and dietary modifications. Oral health education (OHE) intends to improve oral hygiene practices and reduce oral health problems. However, evidence of the impact of multiple topics in OHE on preschool children is lacking. This study aimed to examine the effects of single- versus multiple-topic OHE delivered via video presentations on the plaque and gingival status of preschool children.

Methods

A parallel five-arm cluster randomized controlled trial was conducted on healthy preschool children aged five and six years. Children with chronic illnesses, disabilities, or conditions that could affect their oral health or ability to participate in the OHE intervention were excluded. OHE interventions were given to children from eight of the 10 classes assigned as intervention groups, while two classes served as the control group and received no intervention. The intervention groups received one, two, or three oral health topics using specially developed animation videos, in Malay language: toothbrushing technique (T), toothbrushing technique and the effects of sugar consumption on oral health (TS), toothbrushing technique and pathogenesis of dental caries and gingivitis (TP), and toothbrushing technique, the effects of sugar consumption on oral health, and pathogenesis of dental caries and gingivitis (TSP). Plaque and gingival scores, along with oral health knowledge, attitude, and skills (KAS), were recorded before and after the intervention. The CONSORT guidelines were followed in reporting. The analyses included descriptive statistics, one-way ANOVA, effect sizes, and multivariate analysis of covariance (MANCOVA) at a 5% significance level.

Results

A total of 160 participants were equally distributed into five groups (n = 32). There were no baseline differences in plaque or gingival scores. All groups showed significantly lower plaque and gingival scores post-intervention (p < 0.05), with effect sizes ranging from -1.1 to -0.7. No changes in oral health (KAS) were observed. The intervention groups had significantly lower plaque and gingival scores compared to the control group (p < 0.05), but no differences were found between them after adjusting for baseline KAS (p > 0.05).

Conclusions

This study suggests that including multiple topics in OHE programs for preschool children may not necessarily improve oral health outcomes. Simplified OHE may be more advantageous in terms of time, cost, human resources, and organization.

## Introduction

Dental caries is a microbial infection resulting in the demineralization of the hard tissues of the teeth, leading to cavity formation. Dental caries is a preventable disease that remains highly prevalent among children globally and, while transmissible through the transfer of oral bacteria such as Streptococcus mutans typically from mother to child, differs from infectious diseases that are spread through direct contact, air, or vectors; unlike communicable diseases, dental caries can be effectively prevented through proper oral hygiene practices and dietary modifications [1]. Gingivitis involves inflammation and infection of the tissues supporting the teeth, which can result in tooth loss if untreated. Gingivitis, along with dental caries, is a significant public health issue among children due to its high prevalence throughout the globe [2]. Untreated dental caries and gingivitis can lead to physical pain, eating and sleeping difficulties, poor social and school performance, and a reduced quality of life for children [3]. They also increase the burden of care for guardians and the oral healthcare system [4]. One approach to addressing this problem is through oral health education (OHE).

The strategies of past intervention programs were presented as a package of multiple topics, delivered through multiple delivery methods, including one or more target groups [5]. However, evidence on the effectiveness of current OHE interventions for improving children’s oral health remains debatable. Systematic reviews indicate no meaningful long-term reduction in plaque levels or the incidence of dental caries and gingivitis following educational interventions, despite improvements in knowledge and attitudes [6,7]. Simplified strategies may be more effective than complex ones, focusing on basic education that is straightforward and easier to understand and follow [8].

A scoping review revealed that past education intervention programs included two to six topics, but there is no evidence or recommendation specifying the optimal number of topics for effective interventions [5]. Single-topic interventions focus on specific aspects of oral health, while multiple-topic interventions provide comprehensive education covering various aspects. However, implementing multiple-topic approaches may overwhelm young children, making it difficult for them to process and retain the information [9]. To date, there is no known published study on the impact of the number of topics in OHE.

This study compared the efficacy of OHE in improving plaque and gingival status in preschool children receiving single, double, and triple OHE topics via animation video presentations. It was hypothesized that single-topic OHE is as effective as multiple-topic OHE in improving the oral health status of preschool children, partly due to enhancements in their oral health knowledge, attitudes, and skills (KAS). The findings may help oral healthcare providers and policymakers design and strategize effective OHE programs.

## Materials and methods

Design and ethical consideration

A parallel five-arm cluster randomized controlled trial with an allocation ratio of 1:1:1:1:1 was employed. The study protocol was approved by the USM Human Research Ethics Committee (USM/JEPeM/KK/23060506) and registered with ClinicalTrials.gov (NCT06073392) [10]. Approval was obtained from the preschool administrator, and consent was obtained from the guardians. The study adhered to the Helsinki Declaration, and the report followed the CONSORT guidelines [11].

Participants, settings, and location

Participants included five- and six-year-old preschool children in Kota Bahru, Kelantan. Healthy children, who understood the Malay language and could follow instructions, were included. Children with no chronic illnesses, disabilities, or conditions that could affect their oral health or ability to participate in the OHE intervention were excluded.

Interventions

A separate animation video containing information on a toothbrushing technique, the effects of sugar consumption on oral health, and the pathogenesis of dental caries and gingivitis was specially developed for the intervention. This animation video was created specifically for this study, adhering to guidelines and considering the cognitive abilities of preschool children [12]. The language of instruction was Malay, ensuring that the materials were accessible and understandable for the preschool children involved. For groups receiving more than one topic, the videos were combined. The topics in the video were tailored to match the items in the oral health KAS questionnaire.

The video on toothbrushing, lasting 3.5 minutes, describes the plaque removal technique, toothbrushing frequency, and the use of fluoridated toothpaste. The video on the effects of sugar consumption on oral health, lasting 4 minutes, explains the frequency and duration of sugary intake and the types of sugar in their diet. The video on the pathogenesis of dental caries and gingivitis, lasting 4.5 minutes, describes how the disease develops from dental plaque accumulation. The OHE message was delivered in a storytelling format, featuring interactions between two siblings, their mother, and friends discussing related oral health issues at home (toothbrushing and sugar), school, and the dental clinic (pathogenesis). The OHE video was presented for five consecutive days, at the same time each morning, in a dedicated room at the school, using a projector, screen, and external speakers.

Before data collection, consent forms and questionnaires on sociodemographic information were distributed to the guardians. Oral screenings and assessments of oral health (KAS) were conducted on the selected study participants before administering the respective interventions. The same procedure was repeated two weeks after the interventions.

Oral screenings were carried out using a portable dental chair, LED lights, and disposable probes and mirrors in a dedicated room. The plaque index and gingival index scores were assessed following the method described by Silness and Löe [13,14], respectively, on index teeth (16, 55-51, 61-65, 26, 36, 75-71, 81-85, 46), at four surfaces (distal, mesial, buccal, or labial, and palatal or lingual). The total plaque score and gingival score ranged from 0 to 288. All data were recorded using a dental charting sheet adapted from the World Health Organization [15]. The clinical data were collected by a calibrated clinician experienced in data collection for national surveys of schoolchildren.

The oral health KAS was assessed in groups of five children at a time. They were given a set of questionnaires to complete in 15 minutes and used colored stickers to mark their answers while the researcher read the questions and instructed them to choose their responses.

Outcome assessment

Primary Outcome

The primary outcomes were the plaque and gingival scores at baseline and after intervention.

Secondary Outcome

The secondary outcomes were the oral health KAS adapted from earlier studies [16-19]. There were two questions each for KAS about toothbrushing, the effects of sugar consumption on oral health, and the pathogenesis of dental caries and gingivitis, giving a total of six questions for each topic (Appendix A). Cronbach’s alpha for the respective questions of each topic ranged from 0.4 to 0.8, with test-retest reliability (intraclass correlation coefficient) from 0.59 to 0.85. Each question on KAS was in a closed-ended form. The responses were recorded as “Yes” and “No,” with scores of one and zero given for each correct and incorrect answer, respectively. The correct answers were totaled to give the overall score for KAS, which ranged from 0 to 18.

Sample size determination

A sample size calculation was carried out considering a mean difference between the intervention groups of 0.22 (SD = 0.64), an effect size (ES) of 0.5, 5% alpha error, and 80% study power [20]. Calculation in G*Power version 3.1.9.2 software showed that 133 subjects were needed in total. A total of 160 subjects was planned, accounting for a 20% dropout rate.

Randomization, allocation, and blinding

There were five classes for participants aged five and six years. Cluster randomization was used to allocate two classes from each age group to receive one, two, or three topics: toothbrushing technique only (T), toothbrushing technique and the effects of sugar consumption on oral health (TS), toothbrushing technique and pathogenesis of dental caries and gingivitis (TP), and toothbrushing technique, the effects of sugar consumption on oral health, and pathogenesis of dental caries and gingivitis (TSP). A control group received no intervention. The sample was stratified by class and sex to ensure an equal number of participants in each group and then selected using a simple random sampling method. A single blinding of the children and their families was adopted.

Statistical analysis

Descriptive analysis was used to describe the sample and measures. A one-way ANOVA was used to compare changes in the outcome measures between the groups, with post-hoc comparisons using Games-Howell corrections. Multivariate analysis of covariance (MANCOVA) was used to concurrently compare changes in plaque and gingival scores between the groups and adjust for the baseline oral health KAS scores using bootstrapping with 1,000 iterations. Both intention-to-treat (ITT) and per-protocol (PP) analyses were performed according to the CONSORT principles. The effect size was calculated using Cohen’s criteria: 0.1 = low effect, 0.3 = medium effect, and 0.5 = large effect. All comparisons were analyzed at a 5% significance level in SPSS Version 27.0 (IBM Corp., Armonk, NY).

## Results

Sociodemographic profile

**Figure 1:**
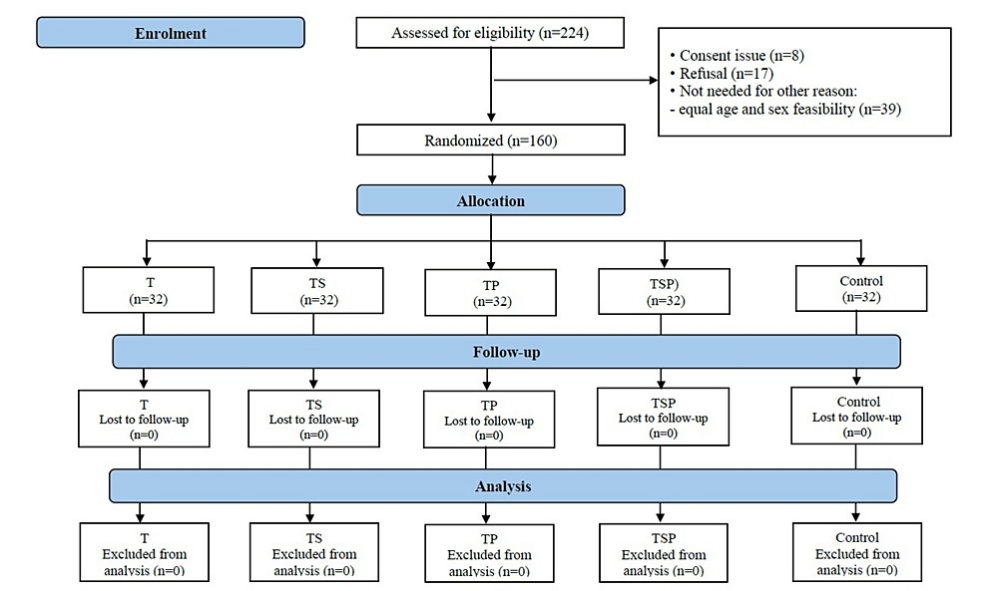
Flow of participants during the study period T, toothbrushing technique; TS, toothbrushing technique and sugar; TP, toothbrushing technique and pathogenesis of dental caries and gingivitis, TSP, toothbrushing technique, sugar, and the pathogenesis of dental caries and gingivitis

A total of 224 participants were screened, and 160 were selected for the study with no loss of participation (Figure 1).

There were 32 participants per group: 128 subjects (80.0%) in the intervention groups and 32 subjects (20.0%) in the control group (Table 1).

**Table 1:** Sociodemographic profile of preschool children (n = 160) ^a^Mean (SD) T, toothbrushing technique; TS, toothbrushing technique and sugar; TP, toothbrushing technique and pathogenesis of dental caries and gingivitis; TSP, toothbrushing technique, sugar, and the pathogenesis of dental caries and gingivitis

Variables	T (n=32), n (%)	TS (n=32), n (%)	TP (n=32), n (%)	TSP (n=32), n (%)	Control (n=32), n (%)
Age (years)^a^	5.5 (0.51)	5.5 (0.51)	5.5 (0.51)	5.5 (0.51)	5.5 (0.51)
Sex					
Boy	16 (50.0)	16 (50.0)	16 (50.0)	16 (50.0)	16 (50.0)
Girl	16 (50.0)	16 (50.0)	16 (50.0)	16 (50.0)	16 (50.0)
Race					
Malay	32 (100.0)	32 (100.0)	32 (100.0)	32 (100.0)	32 (100.0)
Guardian education level					
Secondary school	15 (46.9)	17 (53.1)	16 (50.0)	13 (40.6)	9 (28.1)
College/university	17 (53.1)	15 (46.9)	16 (50.0)	19 (59.4)	23 (71.9)
Guardian monthly household income					
Less than RM2,000.00	5 (15.6)	4 (12.5)	5 (15.6)	6 (18.8)	0 (0.0)
RM2,000.00-RM5,000.00	18 (56.3)	20 (62.5)	19 (59.4)	21 (65.6)	16 (50.0)
More than RM5,001.00	9 (28.1)	8 (25.0)	8 (25.0)	5 (15.6)	16 (50.0)

Changes after intervention

All intervention and control groups showed a significant reduction in the mean plaque and gingival scores after the intervention (p ≤ 0.05) (Table 2). There was no significant change in the oral health KAS scores. The greatest reduction in plaque score after the intervention was in the TP group, while the lowest reduction was in the control group. Post-hoc analysis showed that the difference was significant between all intervention groups and the control (p < 0.05). The greatest reduction in gingival score after the intervention was in the T and TP groups, while the lowest reduction was in the control group. Post-hoc analysis showed that the difference was significant between the T and TP groups and the control (p < 0.05).

**Table 2:** Changes in mean plaque, gingival, knowledge, attitude, and skill scores at baseline and after intervention between groups of preschool children (n=160) ^a^Post hoc (change after intervention) mean difference (SE): T vs C = -5.3 (1.64) (p = 0.013), TS vs C = -3.2 (1.12) (p = 0.044), TP vs C = -5.7 (1.40) (p = 0.002), TSP vs C = -4.0 (1.38) (p = 0.048) ^b^Post hoc (change after intervention) mean difference (SE): T vs C = -3.9 (1.19) (p = 0.018), TP vs C = -3.8 (1.26) (p = 0.032) T, toothbrushing technique; TS, toothbrushing technique and sugar; TP, toothbrushing technique and pathogenesis of dental caries and gingivitis; TSP, toothbrushing technique, sugar, and the pathogenesis of dental caries and gingivitis

Variables	T (n=32)		TS (n=32)		TP (n=32)		TSP (n=32)		Control (n=32)		p-value
	Before	After	Before	After	Before	After	Before	After	Before	After	
	Mean	Mean	Mean	Mean	Mean	Mean	Mean	Mean	Mean	Mean	
	(SD)	(SD)	(SD)	(SD)	(SD)	(SD)	(SD)	(SD)	(SD)	(SD)	
	Mean difference (SE)		Mean difference (SE)		Mean difference (SE)		Mean difference (SE)		Mean difference (SE)		
Plaque score	100.8	94.4	107.7	103.2	105.6	98.8	116.0	110.81	111.5	110.31	0.006 ^a^
	(16.65)	(16.32)	(12.46)	(11.97)	(16.42)	(17.67)	(16.44)	(17.02)	(15.40)	(15.43)	
	-6.5 (1.31)		-4.5 (1.01)		-6.8 (1.37)		-5.2 (1.35)		-1.2 (0.27)		
Gingival score	102.5	96.4	109.2	105.0	106.9	100.8	116.5	111.2	112.1	109.8	0.032 ^b^
	(17.36)	(16.66)	(12.49)	(12.54)	(16.65)	(17.41)	(15.74)	(16.21)	(15.47)	(14.85)	
	-6.1 (1.14)		-4.2 (0.90)		-6.1 (1.20)		-5.3 (1.08)		-2.2 (0.36)		
Knowledge	6.6	6.2	6.7	6.3	6.6	6.2	6.6	6.3	6.5	6.3	0.3
	(0.71)	(0.42)	(0.99)	(0.58)	(0.67)	(0.42)	(0.76)	(0.55)	(0.57)	(0.64)	
	-0.4 (0.13)		-0.4 (0.13)		-0.4 (0.11)		-0.2 (0.12)		-0.2 (0.12)		
Attitude	7.6	6.8	7.6	6.7	7.7	6.8	7.7	7.0	7.7	6.9	0.8
	(0.67)	(0.59)	(0.67)	(0.59)	(0.47)	(0.69)	(0.54)	(0.62)	(0.63)	(0.71)	
	-0.8 (0.15)		-0.9 (0.13)		-0.9 (0.13)		-0.7 (0.11)		-0.8 (0.13)		
Skills	7.3	6.7	7.2	6.6	7.3	6.5	7.3	6.5	7.2	6.4	0.6
	(0.64)	(0.78)	(0.64)	(0.67)	(0.72)	(0.51)	(0.64)	(0.67)	0.63)	(0.49)	
	-0.6 (0.10)		-0.6 (0.10)		-0.8 (0.12)		-0.8 (0.14)		-0.8 (0.12)		

The effect size of the changes in mean plaque scores for the respective interventions ranged from good to excellent, between -0.7 and -0.9. For gingival scores, the effect size was excellent, ranging from -1.1 to -0.8. There was no significant difference in the mean change in oral health KAS scores across the groups (Table 3).

**Table 3:** Effect size of the change in mean plaque and gingival scores at baseline and after intervention (n=160) T, toothbrushing technique; TS, toothbrushing technique and sugar; TP, toothbrushing technique and pathogenesis of dental caries and gingivitis; TSP, toothbrushing technique, sugar, and the pathogenesis of dental caries and gingivitis

Variables	Groups	Point estimates	95% CI
Plaque score	T	-0.9	-1.258, -0.452
	TS	-0.7	-1.098, -0.327
	TP	-0.9	-1.272, -0.462
	TSP	-0.7	-1.046, -0.285
	Control	-0.8	-1.164, -0.378
Gingival score	T	-0.9	-1.349, -0.521
	TS	-0.8	-1.213, -0.417
	TP	-0.9	-1.279, -0.468
	TSP	-0.9	-1.248, -0.444
	Control	-1.1	-1.510, -0.641

MANCOVA was carried out for three reasons: 1) the plaque score was significant at baseline, 2) to consider the changes in plaque and gingival scores concurrently, and 3) to account for KAS scores at baseline. The results were similar to the ANOVA analysis for the plaque (p = 0.006) and gingival scores (p = 0.034). However, for the latter, TSP was significantly different from the control group (p = 0.005) (Table 4).

**Table 4:** Comparison of the change in plaque and gingival scores at baseline and after intervention across groups of preschool children: influence of oral health knowledge, attitude, and skills at baseline (n = 160) Estimate: The estimated mean difference for each group compared to the control group bootSE: Bootstrapped standard error with 1,000 iterations 95% bootCI: The 95% confidence interval from MANCOVA with 1,000 iterations Bias: The bias of the estimate ^a^Significance level from the MANCOVA test ^b^Significance level from the ANOVA test T, toothbrushing technique; TS, toothbrushing technique and sugar; TP, toothbrushing technique and pathogenesis of dental caries and gingivitis; TSP, toothbrushing technique, sugar, and the pathogenesis of dental caries and gingivitis

Variable	Group	Estimate	bootSE	95% bootCI	Bias	p-Value
Plaque score	T	-5.40	1.41	-8.494, -2.475	0.28	0.001^a^
	TS	-3.40	1.14	-5.668, -1.363	-0.08	0.004^a^
	TP	-5.71	1.40	-8.537, -2.918	0.03	0.001^a^
	TSP	-4.10	1.40	-6.941, -1.467	-0.02	0.004^a^
	Control	Reference				0.006^b^
Gingival score	T	-3.82	1.20	-6.267, -1.461	0.02	0.001^a^
	TS	-1.95	1.09	-4.125, -0.088	-0.06	0.072^a^
	TP	-3.87	1.29	-6.662, -1.387	-0.05	0.005^a^
	TSP	-3.05	1.15	-5.407, -1.032	0.00	0.014^a^
	Control	Reference				0.034^b^

## Discussion

The present study investigated the effect of the number of topics in educational videos on improving oral health status and accounting for oral health KAS in preschool children. The study employed four intervention groups exposed to videos featuring different numbers of educational topics and one control group receiving no intervention. The study found that all interventions significantly improved plaque and gingival scores, but there was no difference between the intervention groups. The findings suggest that presenting single or multiple OHE video topics has similar effects on the oral health of preschool children.

Previous research in this domain has predominantly focused on intervention programs presented as a package with overlapping topics delivered using multiple modes of delivery. This pioneering study aims to establish evidence supporting the strategy of delivering multiple topics in OHE for preschool children. The study covered the topics of toothbrushing technique, sugar, and the pathogenesis of dental caries and gingivitis, presenting them using specially developed video animations. Since there is no advantage in presenting multiple topics over a single topic, oral health professionals should consider a simplified strategy when delivering OHE. This strategy can also help lower the cost of intervention materials and manpower resources and shorten the duration of the intervention. In addition, although children aged five and six years are still developing their cognitive abilities, previous studies have shown that age-appropriate educational interventions can effectively improve their oral health knowledge and practices [19,20].

This study found that all groups, including the control group, showed improvement in plaque and gingival status after the intervention. The improvement in oral health was similar to that reported by Wu et al. [21] and Qadri et al. [22], in which school-based programs improved oral hygiene practices following heightened awareness and discussions about oral health in the school environment. The effect of the school program may have spilled over and indirectly benefited the schoolchildren, including those in the control group. The findings may be explained by the Hawthorne effect, where the children altered their behavior because they were aware they were being observed [23]. Nevertheless, the improvement found in the control group of this study was significantly lower compared to the intervention groups. Thus, the additional improvement shown in the intervention groups may be partly explained by the OHE through the animated video.

The lack of significant improvement in gingival status between the TS and control groups is not clear. This study found no improvement in oral health KAS relating to toothbrushing technique, the effects of sugar consumption on oral health, and the pathogenesis of dental caries and gingivitis; hence, these factors are unlikely to explain the lack of difference between the two groups or the improvements observed in other groups. A similar finding was reported by Sahin [24], who observed that patients' oral hygiene habits were not influenced by their knowledge. Since there was no change in the participants' KAS after the interventions, the risk of contamination across the groups is less likely. Nevertheless, the lack of significant difference can be attributed to the limitations of the instruments used to assess oral health KAS, as well as the children's understanding of the topics in the video presentations. The lengthy period required to administer the questionnaire may have caused the children to lose focus and provide inaccurate responses [25]. The use of color stickers may also have introduced bias or misinterpretation due to personal preference [26].

The findings may be influenced by the cognitive level of preschool children, who are in the early stages of cognitive development with limited understanding and skill in processing information [27]. Adding more topics to OHE sessions could have overwhelmed the participants, making it harder for them to understand compared to focusing on a single topic. In this study, the toothbrushing technique was presented first, followed by additional topics. The short attention spans of the children may have caused them to lose focus on the later topics, even though they were repeated for a week [28]. Future research should investigate whether the order of topics could yield different findings. The video, which can be accessed in the public domain (https://youtu.be/qeR91O4Aa80), is suited to children’s cognitive levels and needs for the trial. The animation includes visual and auditory stimuli, which help young learners retain information [29].

Limitations of the study

Apart from the limitations already mentioned, this study is further constrained by the short-term study period and localized population; therefore, the findings should be interpreted with caution. While it is true that children in this age group are still developing their intellectual capacities, studies have shown that they can benefit from well-designed OHE interventions. Short-term effects can be significant as children are capable of retaining information for about two weeks, which is sufficient for initial behavior changes [30].

## Conclusions

This study showed that presenting a single topic, compared to multiple topics, has similar effects on improving the oral hygiene status of preschool children and is not influenced by KAS level. The findings suggest a reorientation of the strategy toward a simpler approach to educating young children about oral health. Future research should investigate similar research questions in different age groups, combinations with other topics, different sequences of topics, and other methods of delivery.

## Appendices

Appendix A

OH Knowledge (Part B Questionnaire):

i.          Dental plaque is a white smear.

ii.         Dental plaque causes caries and gingivitis.

iii.        Sugary food causes caries.

iv.        Healthy food is good for teeth and gums.

v.         Toothbrushing can remove plaque.

vi.        Fluoride strengthens teeth.

OH Attitude (Part C Questionnaire):

i.          Perceived risk of caries and gingivitis.

ii.         Dental check-ups are important.

iii.        Love sweet food.

iv.        Love healthy food.

v.         Own a toothbrush.

vi.        Use fluoride toothpaste.

OH Skills (Part D Questionnaire):

i.          Dental check-up every year.

ii.         Use pea-sized toothpaste.

iii.        Sweet food at school.

iv.        Sweet food at home.

v.         Brush teeth before sleep.

vi.        Guardian helps brush teeth.
